# The Therapeutic Effect of Active Vitamin D Supplementation in Preventing the Progression of Diabetic Nephropathy in a Diabetic Mouse Model

**DOI:** 10.1155/2020/7907605

**Published:** 2020-11-26

**Authors:** Nakhoul Nakhoul, Tina Thawko, Evgeny Farber, Inbal Dahan, Hagar Tadmor, Rola Nakhoul, Anaam Hanut, Ghasan Salameh, Ibrahim Shagrawy, Farid Nakhoul

**Affiliations:** ^1^The Diabetes & Metabolism Lab, Baruch Padeh Poriya Medical Center, Lower Galilee, Israel; ^2^Ophthalmology, Baruch Padeh Poriya Medical Center, Lower Galilee, Israel; ^3^Nephrology & Hypertension Division, Baruch Padeh Poriya Medical Center, Lower Galilee, Israel; ^4^Szeged Faculty of Medicine, Szeged, Hungary; ^5^Nephrology & Hypertension Division, Bar-Ilan University, Ramat Gan, Israel; ^6^Pathology Division, Baruch Padeh Poriya Medical Center, Lower Galilee, Israel; ^7^Azrieli Faculty of Medicine, Bar-Ilan University, Ramat Gan, Israel

## Abstract

**Background:**

Diabetic nephropathy (DN) is one of the most common microvascular complications of diabetes and is the leading cause of end-stage renal disease (ESRD) and replacement therapy worldwide. Vitamin D levels in DN patients are very low due to the decrease in the synthesis and activity of 1-*α* hydroxylase in the proximal tubule cells and decrease in the vitamin D receptor abundance. To date, few studies have shown the antioxidant effects of 1*α*,25-dihydroxyvitamin D_3_ [1,25(OH)_2_D_3_] on hyperglycemia-induced renal injury. The selective activator of the vitamin D receptor, paricalcitol, reduces proteinuria and slows the progression of kidney injury. The precise mechanism through which vitamin D affects diabetic status and provides kidney protection remains to be determined.

**Methods:**

Diabetes mellitus (DM) was induced in 94 8-week-old DBA/2J mice by intraperitoneal injection of streptozotocin (STZ). DM mice were randomly divided into receiving vehicle or treatment with paricalcitol, the active vitamin D analog, 1 week after DM induction or paricalcitol treatment 3 weeks after DM induction. An additional control group of healthy wild-type mice was not treated. Urine albumin, blood urea nitrogen, and creatinine levels were measured before and at the end of the paricalcitol treatment. Periodic acid-Schiff, immunohistochemistry staining, and western blot of the renal tissues of vitamin D receptor, villin, nephrin, and podocin expressions, were analyzed.

**Results:**

Paricalcitol treatment restored villin, nephrin, and podocin protein levels that were downregulated upon DM induction, and reduced fibronectin protein level. Vitamin D receptor activation by paricalcitol may reduce proteinuria of DN in mice and alleviate high-glucose-induced injury of kidney podocytes by regulating the key molecules such nephrin-podocin.

**Conclusions:**

Paricalcitol treatment was associated with improved structural changes in type 1 diabetic mice including upregulation of vitamin D receptor expression, and decreased fibrosis markers such as fibronectin. These effects may contribute to the consistent benefit of vitamin D analog to slow the deterioration in glomerular function and reduce the risk of ESRD in patients with type 1 and 2 diabetes mellitus. Our results suggest that additional use of paricalcitol may be beneficial in treating patients with diabetes under standard therapeutic strategies.

## 1. Introduction

Diabetic nephropathy (DN) is the most common renal complication of uncontrolled type 1 and type 2 diabetes mellitus (T2D) and the leading cause of end-stage renal disease (ESRD). Approximately one-third of all diabetes mellitus (DM) patients will develop ESRD, necessitating renal replacement therapy within 25 years of DM onset [1]. Long-term prospective and interventional studies have clearly demonstrated that the risk of developing DN is directly related to chronic hyperglycemia with different organ damages and failures including the retina, kidney, heart, and blood vessels [2–4]. Among these complications, DN is the most devastating microvascular complication, causing substantial morbidity and mortality [1].

The main pathological changes in DN include functional changes manifested by hyperfiltration and morphological changes in the glomerulus and proximal convoluted tubule (PCT) cells, with podocyte effusion, mesangial matrix hyperplasia, extracellular matrix (ECM) expansion, tubulointerstitial fibrosis, and glomerular sclerosis, leading to ESRD [5–7].

The pathophysiology leading to the development of diabetic nephropathy follows from the diabetic milieu leading to the generation and circulation of advanced glycation end products (AGEs), elaboration of growth factors, and hemodynamic and hormonal changes. These lead to the release of reactive oxygen species and oxidative stress and inflammatory mediators. Several studies have demonstrated that inflammatory cytokines and proinflammatory factors are closely associated with DN generation and progression [6, 7]. Moreover, it is known that renal fibrosis is caused by renal hemodynamic changes, ischemia, glucose metabolism abnormalities, and an overactive renin-angiotensin-aldosterone system (RAAS) [5, 8, 9–11]. Furthermore, transforming growth factor-*β*1 (TGF-*β*1) and angiotensin II contribute to the process of renal tissue fibrosis [5, 12–16]. The roles of fibronectin, a fibrosis-related protein, and TGF-*β*1 in DN pathogenesis and progression have been the focus of many research efforts [5–7].

The involvement of vitamin D in kidney injury has been extensively studied. The active 1,25(OH)_2_D_3_ binds to the intracellular vitamin D receptor (VDR) to activate vitamin D response elements within target genes. In the kidney, vitamin D is important for maintaining podocyte health and suppressing renin gene expression and inflammation in podocytes. Vitamin D has been suggested to harbor multiple biological activities, among them the potential of vitamin D in the protection against DN. Both animal studies and clinical trials have documented an inverse correlation between low vitamin D levels and DN risk, and supplementation with vitamin D or its active derivatives has been demonstrated to improve endothelial cell injury, reduce proteinuria, attenuate renal fibrosis, and retard DN progression. Vitamin D exerts its pharmacological effects primarily via vitamin D receptor on podocytes, whose activation inhibits the renin-angiotensin system. Deb and his group [13] provide evidence that podocyte VDR signaling protects podocytes from hyperglycemia-induced apoptosis and prevents diabetic nephropathy. The anti-DN benefit of vitamin D can be enhanced when administrated in combination with angiotensin-converting enzyme inhibitors or angiotensin II receptor blockers. Mechanistic studies reveal that pathways relevant to inflammation participate in the pathogenesis of DN, indicated by inhibiting macrophage infiltration, nuclear factor-kappa B (NF-*κ*B) activation, and production of such inflammatory mediators as transforming growth factor-*β* (TGF-*β*) [14, 15].

Increasing evidence indicates that vitamin D and its receptor, vitamin D receptor (VDR), play a vital role in the development and progression of DN [4]. VDR, a member of the nuclear hormone superfamily, is expressed mainly in proximal and distal tubular epithelial cells, podocytes, mesangial cells, and collecting duct epithelial cells [14–16]. Several clinical trials have confirmed the antiproteinuric activity of vitamin D analogs in diabetic patients with CKD. In the kidney, VDR is mainly expressed.

Paricalcitol is a modified form of active vitamin D and a VDR agonist, due to its tissue-selective method of action [17–19]. Paricalcitol has a well-established inhibitory effect on cell proliferation and fibrosis [16]. Several studies have shown that VDR-mediated signal transduction pathways play a crucial role in inhibiting the development of DN, via decrease of inflammatory response, reduction of proteinuria, and prevention of fibrosis. Various nephropathy mouse models suggest that paricalcitol has renoprotective effects, including anti-inflammatory and antifibrotic effects [20, 21]. Paricalcitol can increase the expression of VDR on podocytes, promote the translocalization of VDR to the nucleus, and significantly reverse high-glucose-mediated downregulation of nephrin-podocin, thereby improving podocyte injury and reducing urinary albumin in type 2 DM patients [13, 21]. More, paricalcitol can play as a negative endocrine regulator of the renin-angiotensin system with renal protection [10, 12, 22, 23].

To determine the beneficial effects of the selective vitamin D analoge, paricalcitol, on the diabetic renal changes, caused by hyperglycemia, in 8 weeks old DBA/2J mice, by intraperitoneal (IP) injection ,once daily, for five consecutive days, of freshly prepared streptozotocin (STZ). DBA/2J is more susceptible to develop diabetic nephropathy [15, 26]. The purpose of the study was to determine (1) if the exogenous administration of vitamin D analog, paricalcitol, can slow the progression of DN, (2) if paricalcitol can reduce the renal fibrosis due to hyperglycemia, and (3) if paricalcitol can reduce albuminuria via transcriptionally stimulating the expression of nephrin and podocin, a key slit diaphragm protein synthesized by podocytes [27–29].

## 2. Materials and Methods

### 2.1. Animals and Diabetic Model

71 DBA/2J inbred mice were purchased from Jackson Laboratory (Bar Harbor, ME, USA) and group-housed and maintained on a 12 h/12 h light/dark cycle with ad libitum access to food and water. 71 DBA/2J mice were selected, because they are more susceptible to develop diabetic nephropathy. Animals were treated in accordance with NIH Animal Welfare guidelines, and all procedures were approved by the Azrieli Faculty of Medicine, Bar-Ilan University Institutional Animal Care and Use Committee (IACUC).

### 2.2. Diabetes Induction

DM was induced in 8-week-old DBA/2J mice (*n* = 71) by intraperitoneal (IP) injection, once daily, for five consecutive days, of freshly prepared streptozotocin (STZ) (35 mg/kg body weight, dissolved in 10 mM citrate buffer pH 4.5), a pancreatic islet cell toxin (Gurley et al. 2006). The STZ dose regimen was determined in a pilot calibration prior to the model experiment. Control mice (*n* = 23) were treated with a vehicle of 10 mM citrate buffer. One week after last STZ injection, tail blood glucose levels were measured on two consecutive days, using a glucometer (Accu-Chek, Roche). Mice showing glucose levels > 240 mg/dL for two consecutive days were considered diabetic.

### 2.3. Experiment Design

Mice were randomly divided into four groups: (I) control (CON) mice without treatment (*n* = 23: 12 males (M); 11 females (F)); (II) DM-induced diabetic mice received no treatment (*n* = 23: 11 M; 12 F); (III) DM mice treated with paricalcitol (DM+early P treatment) before the onset of diabetic complications, one week after diabetes induction (*n* = 19: 7 M; 12 F); and (IV) DM mice treated with paricalcitol (DM+late P treatment) after the onset of diabetic complications, i.e., three weeks after diabetes induction (*n* = 29: 14 M; 15 F).

Mice were weighed, and urine samples were manually collected every 4 weeks. Mice were euthanized 12 weeks after the beginning of paricalcitol or vehicle treatment, by ketamine-xylazine injection. The blood was immediately collected, and the kidneys were harvested; one was kept in -80°C liquid nitrogen for biochemistry, and the other was kept in formaldehyde for histology examination. The blood was then centrifuged for serum biochemical analysis. The kidneys were weighed and stored according to histological and biochemistry analysis requirements.

### 2.4. Vitamin D Supplementation

One or three weeks after last STZ injection (groups III and IV, respectively), mice were administered an IP injection of paricalcitol (0.3 *μ*g/kg body weight), dissolved in propylene glycol : DDW (80 : 20). Paricalcitol was administered 3 times per week over a total of 12 weeks.

### 2.5. Periodic Acid-Schiff (PAS) Staining

The fresh kidneys from each mouse were fixed in 4% formaldehyde and embedded in paraffin. Then, 4 *μ*m thick sections were cut and transferred to positive glass slides, and processed for periodic acid-Schiff (PAS) staining. Slides were deparaffinized, rehydrated, and then incubated in 0.5% periodic acid for 30 min, followed by an incubation with Schiff's reagent (HX, 69853033, Millipore, USA) at room temperature, for 1 h in the dark. Counterstaining was performed with Mayer's hematoxylin.

### 2.6. Western Blot Analysis

Renal tissues were homogenized with lysis buffer containing protease (#P8340, Sigma, Israel) and phosphatase inhibitors (#04906837-001, F. Hoffmann-La Roche AG, CH-4070 Basel, Switzerland). Kidney lysates were subjected to sodium dodecyl sulfate-polyacrylamide gel electrophoresis (SDS-PAGE), after which proteins were transferred to nitrocellulose or polyvinylidene difluoride (PDVF) membranes. The membranes were then blocked with 5% dry skim milk (Bio-Rad, USA) in Tris-buffered saline with Tween 20 (TBST) at room temperature for 1 h, washed three times with TBST, and incubated with primary antibodies in 5% dry milk in TBST, at 4°C, overnight. The membranes were washed with TBST and incubated with horseradish peroxidase- (HRP-) conjugated secondary antibody at room temperature, for 35 minutes, followed by TBST washes. Bands were visualized using Clarity Enhanced Chemiluminescence (ECL) Kit (Bio-Rad, USA), and band intensities were analyzed using Image Lab software (Bio-Rad, USA).

### 2.7. Western Blot Antibodies

Primary antibodies used included anti-*β*-actin (1 : 10,000, Sigma, USA), anti-glyceraldehyde 3-phosphate dehydrogenase (GAPDH) (1 : 20,000, EPR 16891, Abcam), anti-VDR (1 : 750 NBP1-51322, Novus Biologicals), anti-villin (1 : 1000, LSC407669, Lifespan Biosciences), anti-nephrin (1 : 400, ab58968, Abcam), anti-podocin (1 : 2000, EPR 13820, Abcam), and anti-fibronectin (1 : 2000, Abcam). Secondary antibody included goat anti-rabbit (1 : 7500, 111-035-144, Jackson ImmunoResearch, USA).

### 2.8. Immunohistochemistry (IHC)

Sections were deparaffinized in xylene and graded alcohol, and then subjected to antigen retrieval with citrate buffer 30%, pH 6 (#ZY0001, Zytomed systems). Nonspecific antibody binding sites were blocked with 3% H_2_O_2_ for 15 min. Sections were then incubated with primary antibodies, diluted in blocking solution (CAS-block, 8120, Invitrogen, UK), at 4°C, overnight. HRP-conjugated anti-rabbit (N-Histofine, Nichirei Biosciences Inc., Japan) was used as secondary antibody. Immunoreactive signals were developed upon incubation with 3,3-diaminobenzidine (DAB, TA-060-HDX, Thermo Scientific, UK). Slides were counterstained with hematoxylin, dehydrated, and mounted with Quick hardening mounting (#03989, Sigma, Germany). Images were captured using an Axio Lab/A1 microscope (20x objective), equipped with camera and analyzed with ZEN software. Exposure times were kept constant for all samples.

Primary antibodies used included anti-VDR (1 : 200 NBP1-51322, Novus Biologicals), villin (1 : 400, LSC407669, Lifespan Biosciences), anti-nephrin (1 : 400, ab58968, Abcam), anti-podocin (1 : 2000, EPR 13820, Abcam), anti-fibronectin (1 : 2000, Abcam), and anti-TGF-*β* (1 : 2000, Abcam).

IHC staining was quantified by Panorama Viewer and Image-Pro (version 7) software, using the hotspot method for the most stained fields in each group; 95% confidence intervals were calculated. The quantification was based on at least 10 fields in each group image taken. The total stained area was calculated based on the ratio of the manually selected stained area to the total area of the field.

### 2.9. Statistical Analysis

All results are reported as the mean ± standard error of the mean (SEM), and all experiments were independently repeated at least three times. Student's *t*-test and ANOVA were performed to compare between the study groups, using GraphPad Prism version 5.00 for Windows (GraphPad Software, La Jolla, California, USA) with a *p* value < 0.05 considered statistically significant.

## 3. Results

### 3.1. Paricalcitol Supplementation Effect on Kidney Index

**Figure 1:**
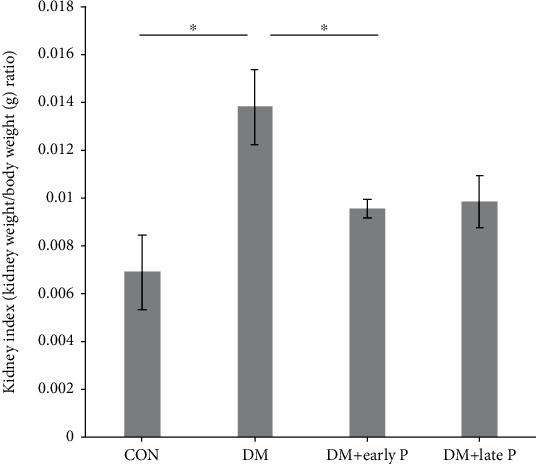
Mouse kidney index. Diabetes induction caused a significant increase in kidney index levels (DM mice, *n* = 13) as compared to control (CON, *n* = 9) mice (^∗^*p* < 0.05), while paricalcitol treatment (DM+early P (*n* = 15), DM+late P (*n* = 12)) restored kidney index levels to normal (*p* < 0.05, DM+early P mice versus control). Results are expressed as the mean ± SEM.

To assess the effect of paricalcitol on kidney function, the kidney index (kidney/body weight ratio) was calculated (Figure 1). The kidney index of DM mice was significantly higher (0.014 ± 0.0016) as compared to control mice (0.007 ± 0.0004) (*p* < 0.05). A moderate decrease in kidney index was shown in DM mice treated with paricalcitol in the late stage of DM (0.01 ± 0.0011), while DM mice receiving early treatment demonstrated significant decreases in the kidney index (0.0097 ± 0.0004) (*p* < 0.05), bringing them to within the normal range.

### 3.2. Paricalcitol Supplementation Effect on Renal VDR Expression

**Figure 2:**
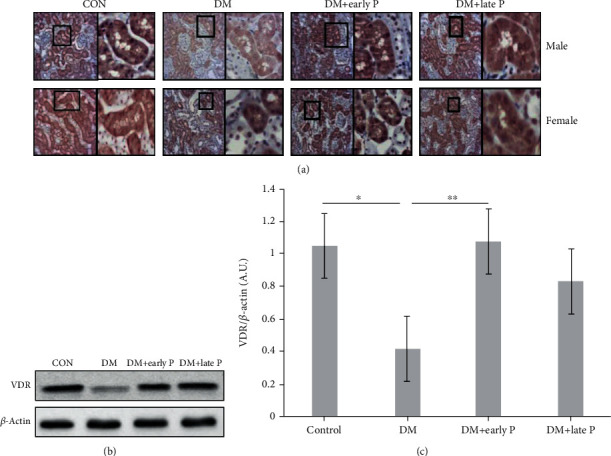
Vitamin D receptor expression in diabetic mouse renal tissues. (a) Immunohistochemical analysis of vitamin D receptor (VDR) expression in mouse renal tissues. VDR signals were lower in DM mice as compared to the control group. Early and late paricalcitol (P) treatments restored VDR expression to normal. Magnification, ×20. (b) Representative western blots of VDR protein levels. (c) Quantification of renal VDR expression. Renal VDR was significantly decreased in DM (*n* = 14) versus control mice (*n* = 13) (^∗^*p* < 0.05). A significant increase in VDR levels was observed in renal tissue of DM+early P (*n* = 16) as compared to DM mice (^∗∗^*p* < 0.01) .

Due to the regulation of VDR levels by active vitamin D levels, the effect of paricalcitol on the renal expression of VDR protein was quantified. While western blot analysis demonstrated a significant decrease in VDR levels in DM mice (0.41 ± 0.1) compared with the control groups (1.05 ± 0.16) (*p* < 0.01), paricalcitol-treated mice (both early and late treatments) expressed VDR (1.07 ± 0.16 and 0.83 ± 0.17, respectively) at levels similar to those of untreated control mice (Figures 2(b) and 2(c)).

These observations were confirmed by IHC staining of VDR in kidney tissues (Figure 2(a)). More specifically, while lower VDR expression was observed in tissues extracted from DM mice as compared to control mice, paricalcitol treatment increased renal VDR levels, with insignificant differences measured between early and late treatments.

### 3.3. The Effect of Paricalcitol on Renal Injury and Morphology

**Figure 3:**
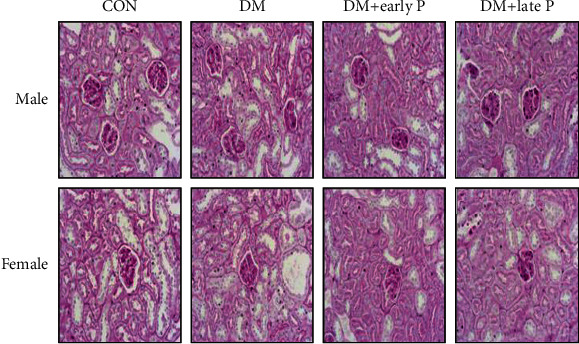
PAS staining of kidney sections. Representative PAS staining of kidney sections shows normal morphology of glomerulus and PCT in the control group. DM mice exhibited extended tubules, while early and late paricalcitol treatments restored the extended tubule shape to normal. Magnification, ×20.

PAS staining of the kidney sections of the control animals revealed normal glomerulus and circular PCT morphologies. In contrast, DM mice exhibited flattened tubular cells. Early and late paricalcitol treatments restored the extended tubule shape to normal (Figure 3).

**Figure 4:**
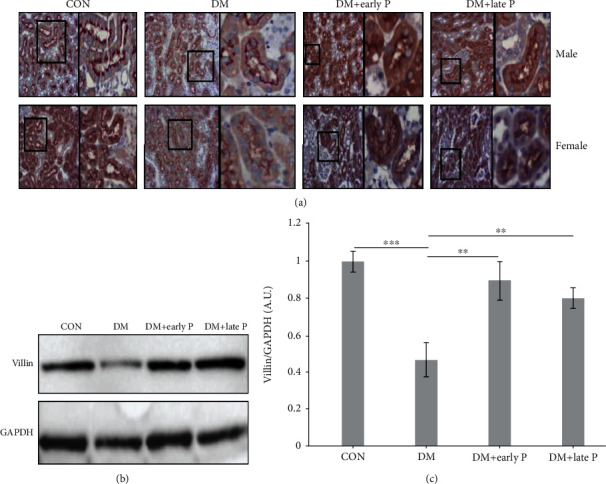
Fibronectin expression in mouse renal tissues. (a) Immunohistochemical analysis of fibronectin expression in diabetes mellitus (DM) mouse kidney sections. Sections of DM mice exhibited high expression of fibronectin as compared to control (CON) sections. Paricalcitol treatment (DM+early P, DM+late P) decreased fibronectin expression. Magnification, ×20. (b) Representative western blots of fibronectin and GAPDH proteins extracted from mouse renal tissues. (c) Quantification of total western blots. Renal fibronectin levels were significantly increased in DM mice (*n* = 10) compared with control mice (*n* = 11) (^∗∗∗^*p* < 0.001). A significant decrease in fibronectin levels was observed in paricalcitol-treated mice (DM+early P (*n* = 10), DM+late P (*n* = 9) treatments) compared with DM mice (^∗∗∗^*p* < 0.001). Error bars correspond to SEM.

DM mice exhibited a higher accumulation of fibronectin in the glomerulus as compared to control mice, an effect which was prevented by paricalcitol treatment, as shown both by IHC (Figure 4(a)) and western blot analysis (Figures 4(b) and 4(c)).

### 3.4. Biomarkers of PCT Injury

#### 3.4.1. PCT Injury

**Figure 5:**
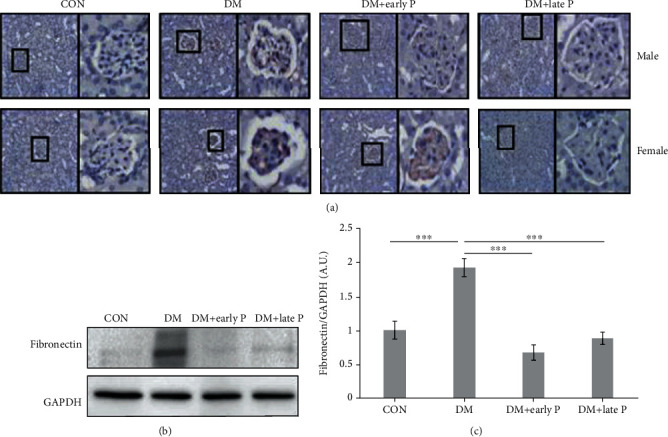
Villin expression in mouse renal tissues. (a) Immunohistochemistry analysis of villin expression in mouse renal sections. Staining intensity of villin was lower in DM as compared to control (CON) mice. Paricalcitol treatment (DM+early P, DM+late P treatments) restored villin levels. Magnification, ×20. (b) Representative western blots of villin and GAPDH proteins extracted from mouse renal tissues. (c) Quantification of total western blots. Renal villin levels were significantly lower in DM mice (*n* = 12) as compared to control mice (*n* = 11) (^∗^*p* < 0.001) and were significantly higher in paricalcitol-treated mice (DM+early P (*n* = 10), DM+late P (*n* = 13) treatments) as compared to DM mice (^∗∗^*p* < 0.01, ^∗∗∗^*p* < 0.01, respectively). Error bars correspond to SEM.

Villin, a major component of the kidney proximal tubule, and a biomarker for PTC injury, was downregulated in DM as compared to control mice (Figure 5(a)), but remained at normal levels in paricalcitol-treated mice. Similar conclusions were reached following IHC analysis of villin protein levels, which were significantly decreased in DM mouse kidneys (0.53 ± 0.09) as compared with control mice (1.14 ± 0.05, *p* < 0.001), but remained at normal levels in mice receiving either early-stage or late-stage paricalcitol treatment (0.92 ± 0.05 and 1.03 ± 0.1, respectively) (Figures 5(b) and 5(c)), suggesting its protective effect on the kidney.

#### 3.4.2. Glomerular Injury

**Figure 6:**
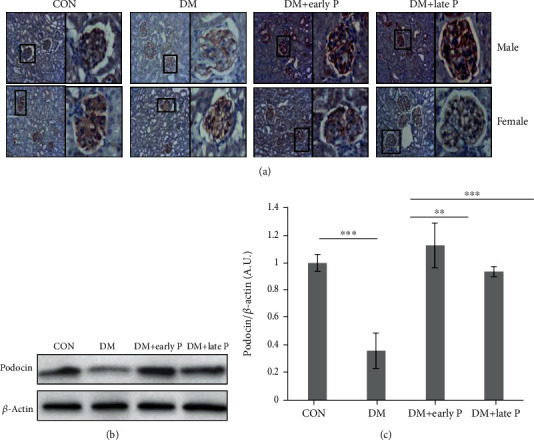
Immunohistochemical analysis of podocin expression in mouse renal tissues. (a) Immunohistochemical (IHC) analysis of podocin expression in mouse kidney sections. Podocin staining was lower in DM as compared to control (CON) mice, while both early and late paricalcitol treatments restored podocin levels. Magnification, ×20. (b) Representative western blots of podocin and *β*-actin proteins extracted from mouse renal tissues. (c) Quantification of total western blots. Renal podocin levels were significantly lower in DM (*n* = 14) as compared to control mice (*n* = 11) (^∗∗∗^*p* < 0.001). A significant increase in podocin levels was observed in paricalcitol-treated (DM+early P (*n* = 10), DM+late P (*n* = 9) treatments) as compared to DM mice (^∗∗^*p* < 0.01, ^∗∗∗^*p* < 0.001, respectively). Error bars correspond to SEM.

Nephrin and podocin, two critical glomerular proteins forming the slit diaphragm, were both downregulated in renal tissues of DM mice as compared to control mice, but levels were restored in paricalcitol-treated animals (Figure 6(a)). Similar findings were obtained following western blot analysis, where podocin levels were lower in DM as compared to control mouse renal lysate (0.3 ± 0.1 and 0.9 ± 0.06, respectively, ^∗^*p* < 0.005), but were normal in paricalcitol-treated animals (1.01 ± 0.09 and 0.87 ± 0.05, early vs. late treatment, respectively) (Figures 6(b) and 6(c)).

**Figure 7:**
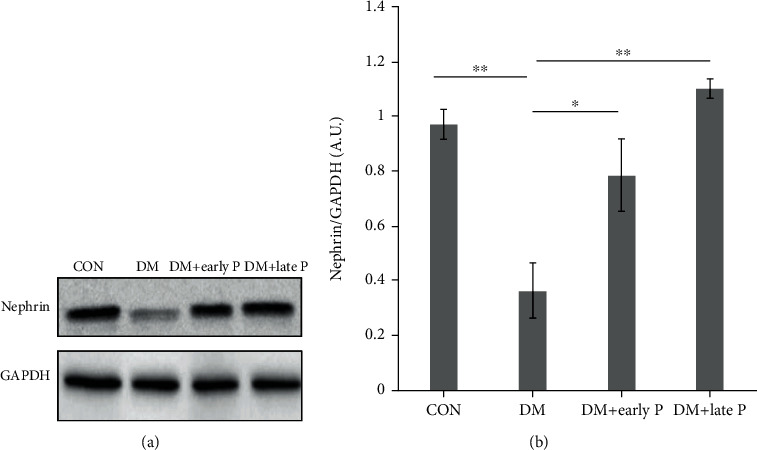
Nephrin levels in mouse renal tissues. (a) Representative western blots of nephrin and GAPDH proteins extracted from mouse renal tissues. (b) Quantification of total western blots. Renal nephrin levels were significantly decreased in DM (*n* = 11) as compared to control mice (*n* = 12) (^∗^*p* < 0.01). Nephrin levels were restored following early (*n* = 12) and late (*n* = 10) paricalcitol treatments as compared to DM mice (^∗^*p* < 0.05, ^∗∗^*p* < 0.01, respectively). Error bars correspond to SEM.

Similarly, while nephrin levels were lower in DM mice (0.5 ± 0.11) as compared with control animals (1.03 ± 0.09), paricalcitol treatment prevented this downregulation (1.26 ± 0.22 and 0.99 ± 0.04, early vs. late treatment, respectively) (Figure 7). Taken together, these results suggest that paricalcitol treatment has a protective effect on PCT and glomerular injury biomarkers.

## 4. Discussion

Although the renoprotective role of vitamin D has been well documented in recent years, the underlying protective mechanism remains elusive. In our study, we provided an evidence that the VDR signaling in podocytes has potent renoprotective activities against diabetic nephropathy, and the podocyte VDR at least in part mediates the antifibrotic action of 1,25(OH)_2_D_3_ and its analogs. More, upregulation of VDR can restore the slit components, nephrin, and podocin damaged by high glucose. The central mechanistic basis of this protection appears to be the inhibition of fibronectin synthesis induced by hyperglycemia. The data reported here strongly support the speculation that podocytes and PCT cells are key renoprotective targets of vitamin D actions.

We assessed the protective role of the vitamin D analog on DN by using DBA/2J inbred mice, inducing diabetes mellitus (DM) by intraperitoneal injection of streptozotocin (STZ). DBA/2J mice are more susceptible to develop diabetic nephropathy.

It is well known that patients with chronic kidney disease are suffering from vitamin D deficiency due to decreased synthesis by the kidney. Vitamin D analog treatment provides significant survival advantages for patients with CKD, as manifested by improvement in renal functions. Several studies have indicated that paricalcitol supplementation can reduce proteinuria in CKD patients [30, 31]. Furthermore, it can decrease podocyte hypertrophy and glomerular sclerosis [32]. However, the mechanism by which paricalcitol protects the kidney and prevents development and progression of DN is poorly understood. The presented data showed that 12 weeks of paricalcitol treatment increased VDR expression in diabetic mouse podocytes can explain the binding mechanism of paricalcitol to VDR and its protective effect on the kidney [21].

The improved mortality rate recorded for paricalcitol-treated DM mice may have been related to VDR stimulation and/or to a direct downregulation of RAAS activity and inhibition of cytokine production, positively affecting survival [24, 25, 33, 34]. Furthermore, vitamin D has physiological functions involving the immune and cardiovascular system and the protection of renal cellular integrity [33].

To investigate the therapeutic effects of paricalcitol on DM-associated proximal tubular injury, we measured the expression of villin, a central component of the cytoskeleton of the epithelial brush borders of the kidney's PCT [35]. The significant upregulation of renal villin expression in diabetic mice receiving paricalcitol treatment suggests that paricalcitol can actively repair renal injury caused by DN. In the kidney, vitamin D is important for maintaining podocyte function and structure [35, 36]. In this study, paricalcitol treatment significantly enhanced nephrin and podocin expression, which had been downregulated upon induction of DM. In addition, a significant increase in VDR expression and restoration of nephrin-podocin proteins in the slit diaphragm was observed in paricalcitol-treated DM mice. These observations align with those of Trohatou et al., who observed enhanced VDR expression on podocytes, localization of VDR in the nucleus, and significant reversal of high-glucose-mediated downregulation of nephrin, thus ameliorating podocyte injury in paricalcitol-treated animals [36]. Similarly, Chokhandre and his colleagues have shown that VDR activation by paricalcitol decreases renal inflammation and podocyte apoptosis in diabetic nephropathy models [17]. Therefore, VDR upregulation in podocytes can be upregulated by the vitamin D supplementation to be renoprotective.

The elevated expression of fibronectin, a potent fibrogenic factor, in diabetic mice as compared with control mice, and its restoration to normal levels following paricalcitol treatment, provided additional evidence of the renoprotective effect of paricalcitol in DM mice. The reported PAS staining results further corroborated these protective effects of paricalcitol on renal fibrosis.

The current study was carried out to explore the potential effect of paricalcitol treatment in preventing the initiation and progression of DN in a STZ-induced diabetes mouse model. According to the presented findings, paricalcitol, a selective and active vitamin D analog, has clear beneficial effects on both renal PCT and glomerular structure and functions, with podocytes likely being a key therapeutic target in vitamin D therapy of diabetic nephropathy. Therefore, the rescue of diabetic renal injury observed in the diabetic mice is a very compelling piece of evidence that supports the importance of the podocyte VDR signaling in renoprotection. Taken together, these data demonstrate podocytes as a key therapeutic target in vitamin D therapy of CKD.

## 5. Conclusions

Paricalcitol could reverse the decrease in the levels of VDR, nephrin, and podocin caused by high glucose, further confirming its effects on prevention of renal injury in DN. These results may provide a basis for further rational use of paricalcitol in the clinic practice in early stages of diabetic nephropathy, and not only in patients with secondary hyperparathyroidism.

## Data Availability

The research data used to support the findings of this study are available from the corresponding author upon request. The corresponding author email is NNakhoul@poria.health.gov.il.
